# Myeloperoxidase inhibition protects bone marrow mononuclear cells from DNA damage induced by the TOP2 poison anti‐cancer drug etoposide

**DOI:** 10.1002/2211-5463.13799

**Published:** 2024-03-26

**Authors:** Ian G. Cowell, Caroline A. Austin

**Affiliations:** ^1^ Biosciences Institute Newcastle University UK

**Keywords:** DNA damage, etoposide, myeloperoxidase, myelotoxicity, TOP2, topoisomerase

## Abstract

Myeloperoxidase (MPO) is found almost exclusively in granulocytes and immature myeloid cells. It plays a key role in the innate immune system, catalysing the formation of reactive oxygen species that are important in anti‐microbial action, but MPO also oxidatively transforms the topoisomerase II (TOP2) poison etoposide to chemical forms that have elevated DNA damaging properties. TOP2 poisons such as etoposide are widely used anti‐cancer drugs, but they are linked to cases of secondary acute myeloid leukaemias through a mechanism that involves DNA damage and presumably erroneous repair leading to leukaemogenic chromosome translocations. This leads to the possibility that myeloperoxidase inhibitors could reduce the rate of therapy‐related leukaemia by protecting haematopoietic cells from TOP2 poison‐mediated genotoxic damage while preserving the anti‐cancer efficacy of the treatment. We show here that myeloperoxidase inhibition reduces etoposide‐induced TOP2B‐DNA covalent complexes and resulting DNA double‐strand break formation in primary *ex vivo* expanded CD34^+^ progenitor cells and unfractionated bone marrow mononuclear cells. Since MPO inhibitors are currently being developed as anti‐inflammatory agents this raises the possibility that repurposing of these potential new drugs could provide a means of suppressing secondary acute myeloid leukaemias associated with therapies containing TOP2 poisons.

## Graphical abstract

TOP2 poisons such as etoposide are widely employed anti‐cancer drugs, but their use is associated with DNA damage that can lead to therapy‐related acute leukaemia. Etoposide is activated by the myeloid‐specific enzyme myeloperoxidase (MPO) to more DNA damaging forms, and we show that MPO inhibition reduces etoposide‐induced DNA damage in bone marrow mononuclear cells in which leukaemias may arise.

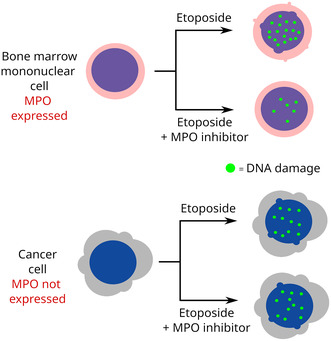

**Abbreviations**
DMSO: dimethyl sulfoxide
DSB: DNA double‐strand break
H2AX: histone H2A.X
TOP2: DNA topoisomerase 2
TOP2A: DNA topoisomerase IIα
TOP2B: DNA topoisomerase IIβ
γH2AX: S‐139 phospho‐histone H2A.X

Improved treatment protocols have resulted in substantially better cancer survival rates. However, it remains a challenge to develop therapies that are less toxic and cause fewer adverse effects than current treatments. Much of the toxicity of cytotoxic chemotherapy involves damage to the bone marrow haematopoietic system. In the acute setting, this includes chemotherapy‐induced neutropenia (reduced number of neutrophils), which is associated with substantial morbidity and mortality and is a common cause of dose reduction and treatment delay. Furthermore, some of the most effective anti‐cancer drugs, including the TOP2 poisons etoposide, doxorubicin, and mitoxantrone, are associated with therapy‐induced acute leukaemia (t‐AL) that can arise years after treatment of the original cancer [1, 2, 3]. TOP2 poisons induce DNA damage in cells by interfering with the reaction cycle of DNA topoisomerase II (TOP2), stabilising a normally transient enzyme‐bridged DNA double‐strand break (DSB). The resulting covalent TOP2‐DNA complexes (TOP2‐CCs) can be converted to protein‐free DSBs (pf‐DSBs) by cellular processing [4, 5, 6]. This activity accounts for the anti‐cancer activity of TOP2 poisons, but also underlies their adverse acute and long‐term effects on haematopoietic cells [3]. Notably, TOP2‐associated t‐AL cases often harbour characteristic balanced chromosome translocations [3] including those involving the *KMT2A/MLL* locus or t(15,17)(*PML‐RARA*), t(8,21)(*AML/RUNX1‐ETO*) and inv(16)(*MYH11‐CBFB*) translocations [7]. These chromosome translocations are believed to occur following DNA DSBs induced by poisoned TOP2 activity [3, 8, 9, 10] and are crucial early events in the development of these therapy‐related leukaemias; the resulting fusion genes, for example *MLL‐AF9*, are able to induce leukaemia in animal models [11, 12]. Therefore, a means of specifically protecting bone marrow haematopoietic progenitors and precursors from the DNA damaging effects of TOP2 poisons would have significant clinical benefit. One potential way of doing this is presented by the limited expression pattern of the haem peroxidase MPO. MPO is expressed almost exclusively in cells of the myeloid lineage and is present at high levels in neutrophils where it has an anti‐microbial function. However, MPO transcripts are also detectable in myeloid precursor/progenitors, including common myeloid progenitor (CMP) cells and are present at high levels in human granulocyte/macrophage progenitor (GMP, CFU‐GM) cells (see Ref. [13] and citations within and BLUEPRINT RNA‐seq data [14]). MPO protein is also readily detectable in *ex vivo* normal human bone marrow CD34^+^ haematopoietic cells [13]. In its physiological role, MPO generates hypochlorous acid (HOCl) from hydrogen peroxide and chloride ions to kill pathogenic microorganisms. However, MPO activity also leads to the oxidation of etoposide catechol, itself a product of cytochrome p450 action, to etoposide quinone that is more effective at inducing TOP2‐mediated DNA breaks than etoposide *in vitro* [13, 15, 16, 17]. Furthermore, etoposide metabolites have the potential to form adducts with DNA and protein, which may also impact on genotoxicity.

Along with its action on etoposide, MPO has also been implicated in the activation of mitoxantrone resulting in its covalent interaction with DNA [18, 19, 20, 21]. Furthermore, reactive aldehydes produced by the action of MPO in the cell [22] can crosslink mitoxantrone and potentially anthracyclines to DNA. In addition, the presence of MPO‐containing cells in the bone marrow compartment may affect surrounding cells via the release of oxidatively activated TOP2 poisons or other reactive compounds into the bone marrow environment. MPO content and activity can be dramatically reduced in cell lines using the heme synthesis inhibitor succinylacetone, but recently a number of potent specific MPO inhibitors have also been developed with a view to their future clinical use [13, 23, 24, 25]. The interest in selective inhibitors is due to the role of MPO in the pathogenesis of a range of disorders including Parkinson's, Alzheimer's, vascular inflammatory disease, and chronic obstructive pulmonary disease [24, 25, 26, 27, 28, 29]. Thus, in principle, the extra unwanted DNA damaging capacity of TOP2 poisons caused by MPO expression in myeloid cells might be avoided by repurposing an already developed MPO inhibitor. In support of this, we have demonstrated that in the MPO‐expressing cell line NB4, MPO inhibition suppresses TOP poison‐induced DNA damage [13]. Furthermore, in conditions of depleted glutathione (which are likely to occur during chemotherapy) MPO inhibition has an even greater effect on etoposide‐induced DNA damage, reducing the level of histone H2AX phosphorylation (a marker of DNA DSBs) by more than 50% [13]. The aim of this study is to determine whether MPO inhibition has the capacity to protect primary human bone marrow mononuclear cells from DNA damage induced by TOP2 poisons. If this is the case, then deploying an MPO inhibitor during cancer chemotherapy could be a practical way of reducing associated toxicities.

While there has been considerable previous work on the ability of MPO to oxidatively activate TOP2 poisons, to our knowledge, there are no other ongoing studies to determine whether MPO inhibitors could reduce bone marrow toxicity associated with chemotherapy regimens.

## Materials and methods

### Reagents and antibodies

Etoposide and PF1355 were purchased from Sigma‐Aldrich (Dorset, UK). PF‐1355 was used at a final concentration of 10 μm, the minimum concentration to achieve near maximal inhibition of MPO in isolated neutrophils [24]. Anti‐MPO ab9535 (immunofluorescence) antibody was from Abcam (Cambridge, UK), anti‐mouse γH2AX 05‐636 was obtained from Merck‐Millipore (Watford, UK). TOP2B mouse monoclonal antibody MAB6348 was obtained from Bio‐Techne (Abbingdon, UK). TOP2A and TOP2B rabbit polyclonal antibodies were sourced in‐house [13].

### Cell culture

Cryopreserved bone marrow CD34^+^ cells immunomagnetically purified from pooled adult bone marrow mononuclear cells were obtained from Stemcell Technologies (Vancouver, British Columbia, Canada). Thawed cells were expanded in Stemspan SFEM II medium with CC100 cytokine cocktail (Stemcell Technologies). For longer term culture the resulting cells were maintained in IMDM + 10% FCS. Cryopreserved unfractionated bone marrow MNCs (Allcells) from single donors were obtained from Caltag (Buckingham, UK, cat # ABM007F: Lot #3007896 – donor 1, M 25yo; Lot #300896 – donor 2, F 27yo) and cultured in IMDM + 10% FCS. Cells were thawed and plated on the day of the experiment according to the supplier's protocol.

### Trapped in agarose DNA immunostaining (TARDIS) assay

TARDIS is a quantitative immunofluorescence‐based method to quantify covalent TOP2‐DNA complexes. Cells were exposed to solvent (DMSO), etoposide or etoposide plus PF1355. In the latter case PF1355 was added first and the cells were preincubated for 4 h before adding etoposide. TARDIS assays were carried out essentially as described previously [30, 31]. Cells were mixed with ultra‐low melting temperature agarose in PBS at 37 °C and spread in a thin layer onto glass microscope slides. After rapid setting of the agarose cells were lysed *in situ* for 30 min in 1% SDS, 20 mm sodium phosphate, 10 mm EDTA pH 6.5 and soluble proteins were extracted by immersing the slides in 1 m sodium chloride for 30 min. Lysis and extraction buffers as well as the following PBS wash buffers contained mammalian protease inhibitor cocktail. The extraction procedure leaves “nuclear ghosts” of genomic DNA in the agarose layer, stripped of protein components apart from any covalently attached molecules. After extensive washing in PBS TOP2 covalent complexes were stained by immunofluorescence using rabbit anti‐TOP2B (4555) antibodies raised to the C‐terminal domain of human TOP2B [13] and AlexaFluor 488 coupled anti‐rabbit antibody (Thermo Fisher Scientific, Altringcham, UK). Slides were counterstained with Hoechst 33258. Hoechst and AlexaFluor images were captured using an Olympus IX‐81 epifluorescence microscope fitted with an Orca‐AG camera (Hamamatsu, Hamamatsu City, Japan) and suitable narrow band filter sets using a 10× objective. Background subtraction and shade correction were carried out as described [31]. After image capture automated slide scoring was performed using volocity 6.3 software (PerkinElmer Inc., Beaconsfield, UK) as described previously [13] using the same parameters for each slide. Data were collated and presented and statistical analysis was performed using graphpad prism 10 (Dotmatics, Boston, USA) and r (https://cran.r‐project.org/).

### Immunofluorescence

Cells were washed and pelleted in ice‐cold PBS and spotted onto poly‐l‐lysine reaction well slides (Marienfeld, VWR, Leicestershire, UK). After allowing cells to settle and attach, they were fixed in 4% formaldehyde in PBS at room temperature. Cells were permeabilised using KCM + T buffer (120 mm KCl, 20 mm NaCl, 10 mm Tris–HCl pH 8.0, 1 mm EDTA, 0.1% Triton X‐100) and blocked in (KCM + T, 2% Bovine Serum Albumin, 10% dry milk powder). Cells were probed with primary and secondary antibodies diluted in blocking buffer. Slides were counterstained with DAPI (Vector Labs, Newark, CA, USA) and viewed using an epifluorescence microscope (Olympus IX‐81). For quantitative integrated fluorescence analysis of γH2AX, imaging was performed as for TARDIS. For γH2AX foci analysis, z‐stacks were captured using a 40× objective and images were scored in extended focus mode using volocity 6.3 software (PerkinElmer Inc.). Data were collated and plotted and statistical analysis was performed using graphpad prism.

## Results

We previously demonstrated that MPO activity increased the appearance of etoposide‐induced TOP2‐DNA covalent complexes and DNA double‐strand breaks, as measured by histone H2AX phosphorylation [13]. These experiments were carried out in established cell lines, NB4 cells (an MPO‐expressing line) and K562 cells (a cell that does not normally express MPO) artificially expressing MPO, and employed MPO inhibitors, including the specific small molecule inhibitor PF1355 [24]. Notably, PF1355 and a second MPO inhibitor MPOi‐II [32] suppressed etoposide‐induced DNA damage in NB4 but not in unmodified K562 cells which do not express MPO [13], arguing against any general DNA damage protecting role of these inhibitors. In the present study, we aimed to examine whether PF1355 can protect *ex vivo* primary cells from etoposide‐induced DNA damage. We used two cell systems to achieve this, (a) *ex vivo* expanded bone marrow CD34^+^ fractionated stem cells derived from bone marrow mononuclear cells (BMCs) and (b) unfractionated BMCs, which are a mixed population of single nucleus cells including immature monocytes and lymphocytes as well as haematopoietic stem and progenitor cells.

### Expression of TOP2A and TOP2B in *in vitro* expanded bone marrow CD34^+^

**Fig. 1:**
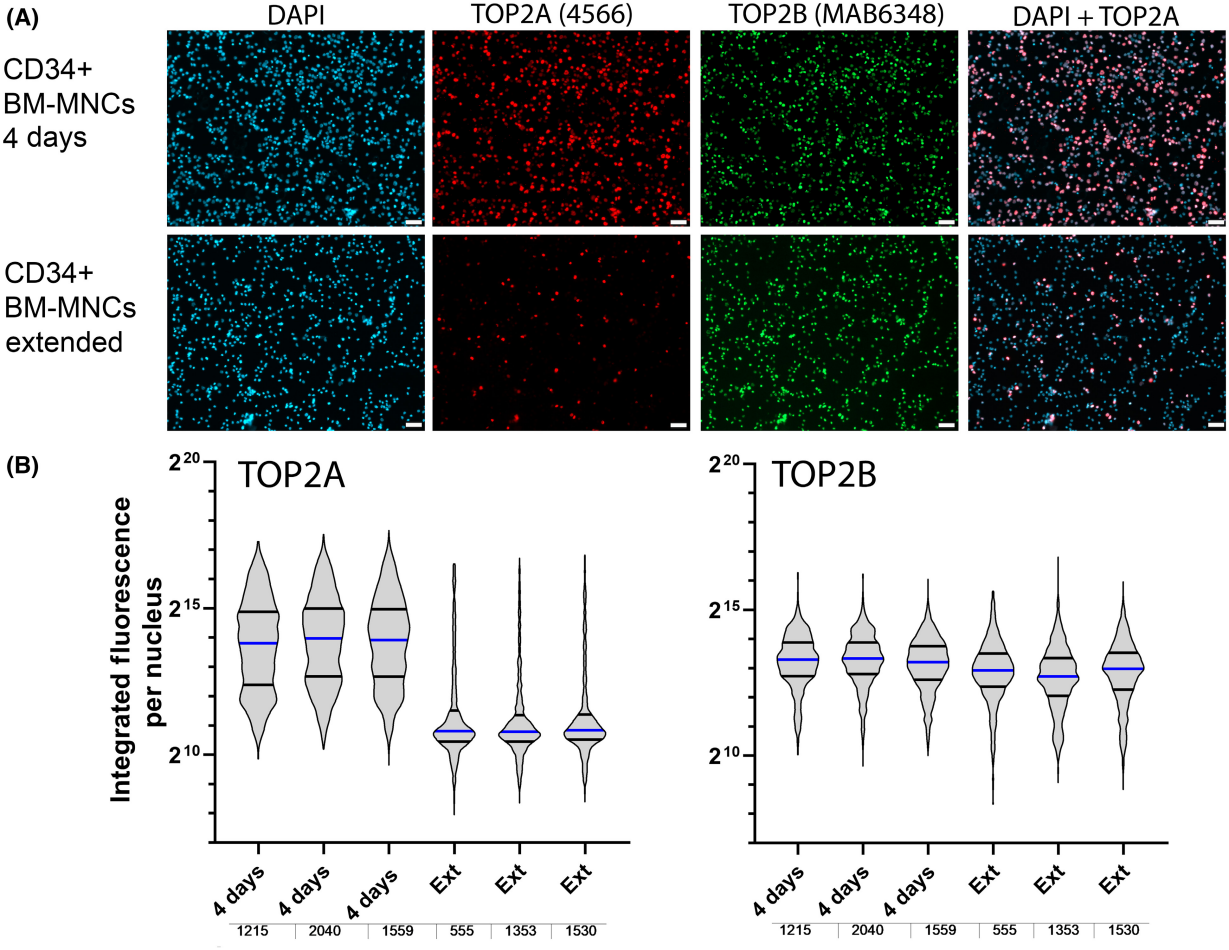
Expression of TOP2A and TOP2B in *ex vivo* CD34^+^ selected bone marrow progenitor cells. CD34^+^ bone marrow MNCs were cultured for 4‐ or 43‐days (Ext) *ex vivo*, and then attached to poly‐l‐lysine‐coated coverslips. Immunofluorescence was performed for TOP2A and TOP2B and images were captured using a 10× objective for quantitative analysis. (A) Representative images. (B) Violin plots illustrating reduced expression of TOP2A and constant expression of TOP2B over extended *ex vivo* culture; blue lines indicate median values. The number of cells quantified in each sample is indicated beneath the graphs. Scale bar = 50 μm.

Vertebrates express two TOP2 isoforms, TOP2A and TOP2B, with very similar *in vitro* activities, but differing *in vivo* functions. TOP2A is essential for cell viability in dividing cells, because of an essential role in cell division. In contrast, TOP2B is not essential for cell viability but is required at the whole organism level [33]. TOP2A is typically downregulated as cells exit the cell cycle, whereas TOP2B expression is maintained even in postmitotic cells. We examined the expression of TOP2A and TOP2B in CD34^+^ fractionated cells that had been cultured (expanded) under conditions to promote stem and progenitor cell proliferation. As shown in Fig. 1A TOP2A was expressed widely, though not uniformly in CD34^+^ cells that had been cultured for a short period (4 days), consistent with the presence of many proliferating cells at this time point. However, at a longer *ex vivo* culture time (43 days) very few cells expressed TOP2A, correlating with loss of stem and progenitor cells due to ongoing *ex vivo* differentiation. This reduced TOP2A expression is also apparent in quantitative immunofluorescence analysis as shown in Fig. 1B. By contrast, TOP2B was expressed more uniformly across the cell population and expression was almost identical at the two time points.

### PF1355 supresses etoposide‐induced DNA damage in *ex vivo* expanded CD34^+^ cells

**Fig. 2:**
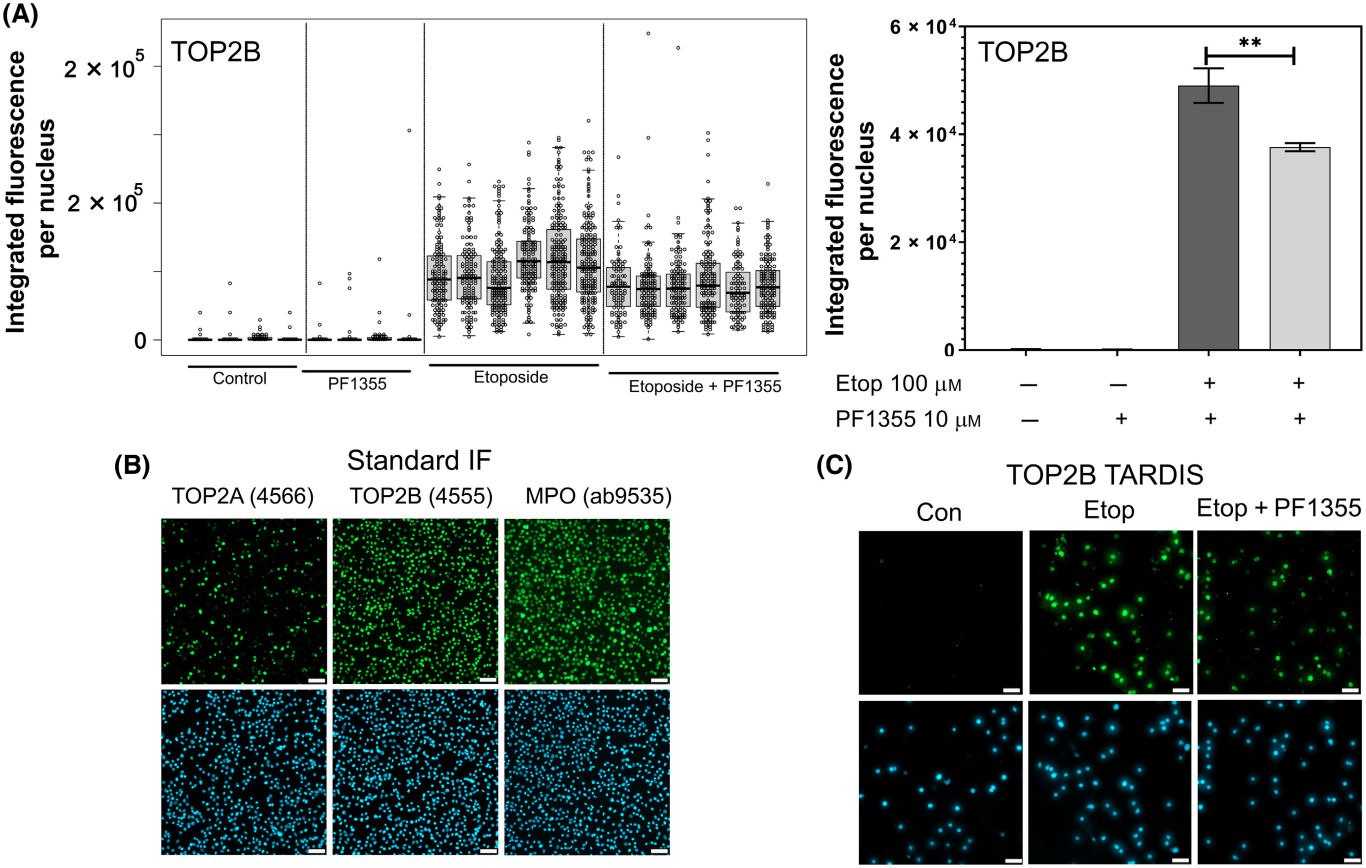
PF1355 reduces the level of TOP2B‐DNA covalent complexes induced by etoposide in expanded CD34^+^ cells. (A) Cells were pre‐treated with PF1355 (10 μm) for 4 h before adding etoposide (100 μm). Cells were incubated with etoposide for 1 h and TOP2B‐DNA covalent complexes were quantified by TARDIS analysis. Integrated fluorescence values were determined per nucleus (at least 500 nuclei per treatment per replicate experiment). Data in the left panel represent the individual integrated fluorescence values per cell for four biological replicates. From these, median values were obtained for each treatment and means of the medians were calculated from replicates and plotted on the right expressed as a percentage of the mean value obtained with 100 μm etoposide in the absence of PF1355 ± SEM. Significance testing was performed by unpaired *t*‐test (**P < 0.01). (B) Standard immunofluorescence analysis of representative samples used for (A) demonstrating the broad expression of TOP2B and MPO. (C) Representative image from the TARDIS data enumerated in (A) and (B). CD34^+^ BMCs were cultured/expanded in SFEM + CC100 for 16 days prior to drug treatment. Scale bar = 50 μm.

TOP2 protein‐DNA covalent complexes (TOP2‐CCs) can be specifically detected and quantified using the quantitative immunofluorescence‐based TARDIS method [13, 30, 31]. In this assay, non‐covalently bound proteins including chromatin proteins such as histones and TOP2 when not complexed with DNA are fully extracted from agarose‐embedded cells using detergent and salt. This leaves nuclear “ghosts” of genomic DNA with any covalently attached proteins trapped in the agarose. Retained TOP2‐CCs are then measured by quantitative immunofluorescence. Figure 2A illustrates the results of such an experiment, involving five replicate samples where cells were treated with etoposide or a combination of etoposide and PF1355. We focussed on TOP2B due to its more widespread and consistent expression compared to TOP2A (see Fig. 1). Co‐treatment with PF1355 consistently reduced the TOP2B‐CC immunofluorescent signal, and when the means of the median values from the five replicates were calculated, this reduction was statistically significant (Fig. 2A). Representative images using standard paraformaldehyde‐fixed immunofluorescence and TARDIS are shown in Fig. 2B,C respectively.

**Fig. 3:**
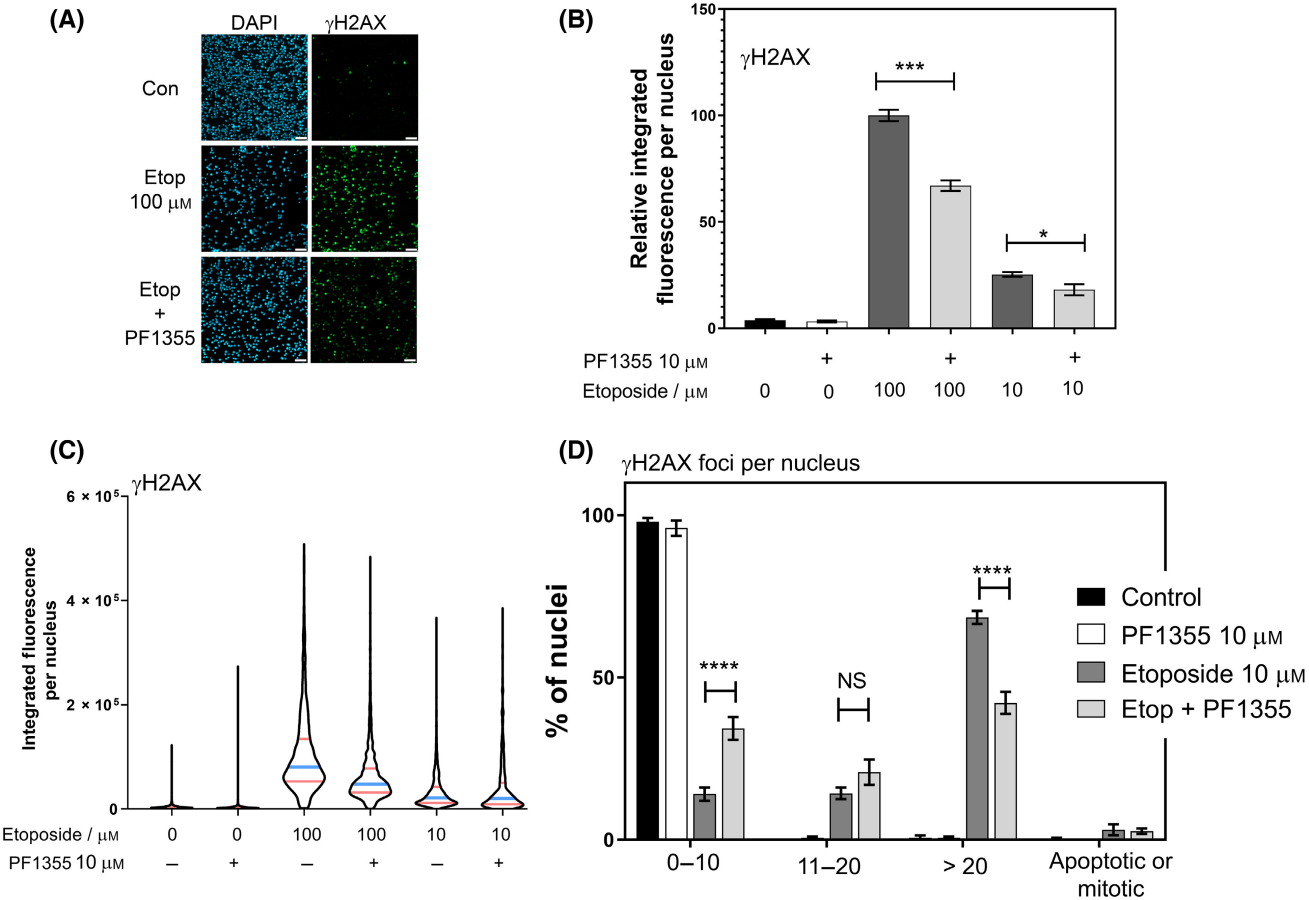
PF1355 suppresses etoposide‐induced DNA damage in *ex vivo* expanded CD34^+^ BM‐MNCs. (A) Expanded CD34^+^ cells were treated with the drug combinations shown, PF1355 (10 μm) pre‐treatment was for 4 h before adding etoposide. Cells were incubated with etoposide for 1 h, before attaching to poly‐l‐lysine‐coated coverslips and immunofluorescent staining for γH2AX. (B) Cells were treated as indicated, attached to poly‐lysine‐coated coverslips and H2AX phosphorylation was quantified by immunofluorescence. Data shown is the mean ± SEM of the median integrated fluorescence per nucleus obtained from four replicas. Statistical testing was by 1‐way ANOVA. (C) Violin plots illustrating the distribution of integrated fluorescence per nucleus values from one of the replicas employed in (B). (D) Analysis of γH2AX foci induced by etoposide and the effect on this of PF1355. Images were captured using a 40× objective and nuclei were placed into bins based on the number of γH2AX foci present. Data are the mean values obtained from three replicas ± SEM. Significance testing was performed using 2‐way ANOVA (Tukey; *P < 0.05, ***P < 0.001, ****P < 0.0001). CD34^+^ BMCs were cultured/expanded for 11 days in culture SFEM+CC100 prior to drug treatment. Scale bar = 50 μm.

TOP2‐CCs can be processed to protein‐free double‐strand breaks (pf‐DSBs) that result in H2AX phosphorylation. In Fig. 3 we show that PF1355 can suppress the level of H2AX phosphorylation per cell (Fig. 3A–C). Enumeration of the numbers of etoposide‐induced γH2AX foci, reflecting the numbers of DSBs per cell, also showed a reduction in the presence of PF1355 (Fig. 3D). Specifically, we placed nuclei into categories based on foci number bins and we observed a significant increase in the number of nuclei exhibiting 0–10 foci and a decrease in the number of nuclei with > 20 foci in cells treated with PF135 and etoposide compared to etoposide alone.

### Expression of TOP2A, TOP2B and myeloperoxidase in bone marrow mononuclear cells

**Fig. 4:**
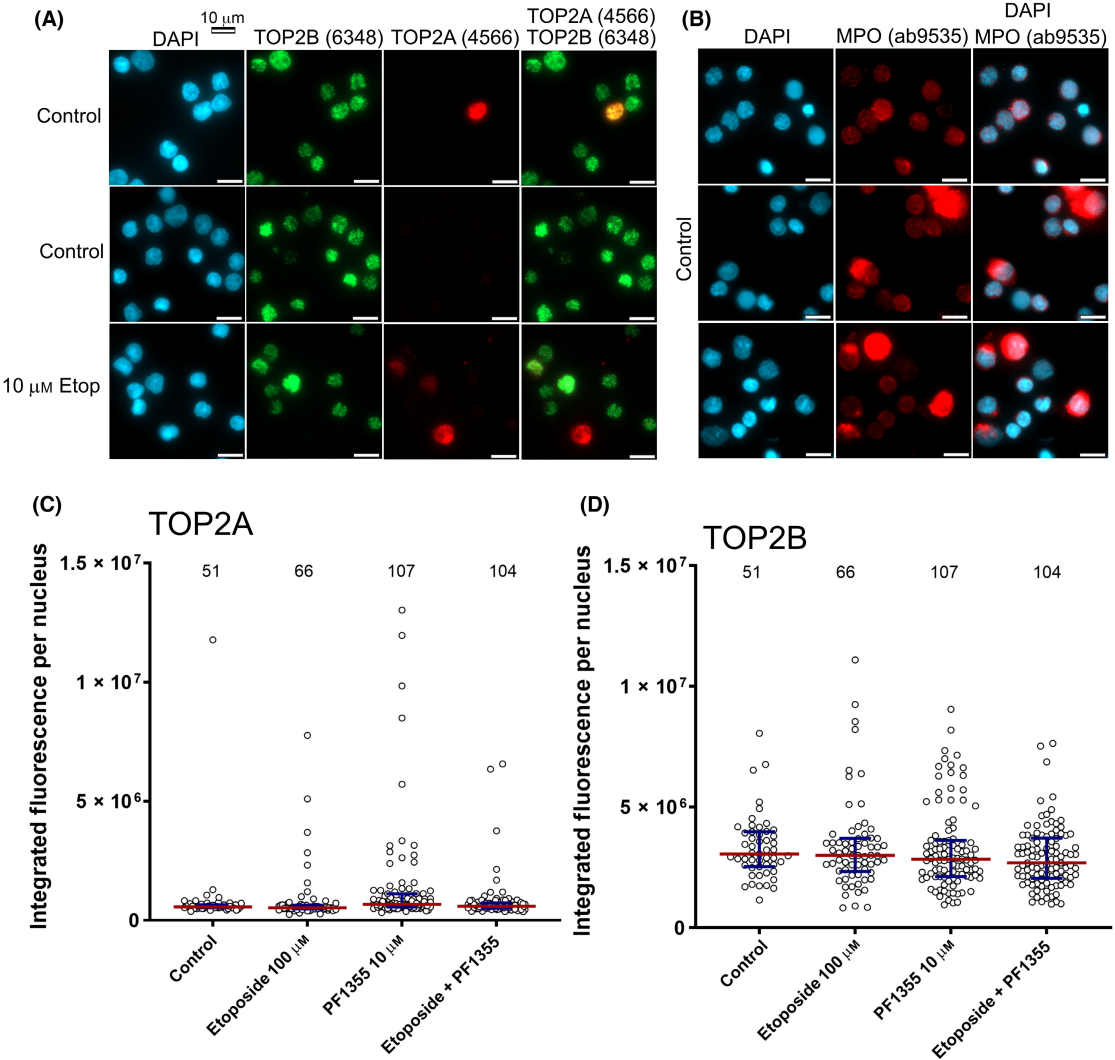
Expression of TOP2 and MPO in bone marrow MNCs. Cryopreserved bone marrow MNCs were thawed and cultured overnight before attaching to poly‐l‐lysine‐coated coverslips and performing immunofluorescence for TOP2A and TOP2B (A) or myeloperoxidase (B). Relative levels of TOP2A and TOP2B expression per cell were determined using quantitative immunofluorescence (C, D). The number of cells included in the quantification are indicated above the graphs. Scale bar = 10 μm.

We examined the expression of TOP2A and TOP2B as well as MPO in unfractionated bone marrow mononuclear cells. These cells were thawed from vials of cryopreserved cells and cultured overnight before attaching to poly‐l‐lysine‐coated coverslips for immunofluorescent analysis. Consistent with the heterogenous nature of the cell types present, and the relatively low number of proliferating cells predicted to be present, we found low level expression of TOP2A in most cells, but with a minority expressing TOP2A at a higher level. In contrast, TOP2B expression was detected in almost all cells (Fig. 4A). Interestingly, most cells expressed MPO moderately, and in a minority of cells, MPO was present at a high level (Fig. 4B). Nuclear expression levels of TOP2A and TOP2B were confirmed by quantitative immunofluorescence (Fig. 4C,D). Notably, the expression of neither TOP2A nor TOP2B was affected by incubation of the cells with Etoposide (10 μm, 6 h), PF1355 (10 μm, 24 h) or both combined (Fig. 4C,D).

### PF1355 supresses etoposide‐induced DNA damage in *ex vivo* cultured bone marrow mononuclear cells

**Fig. 5:**
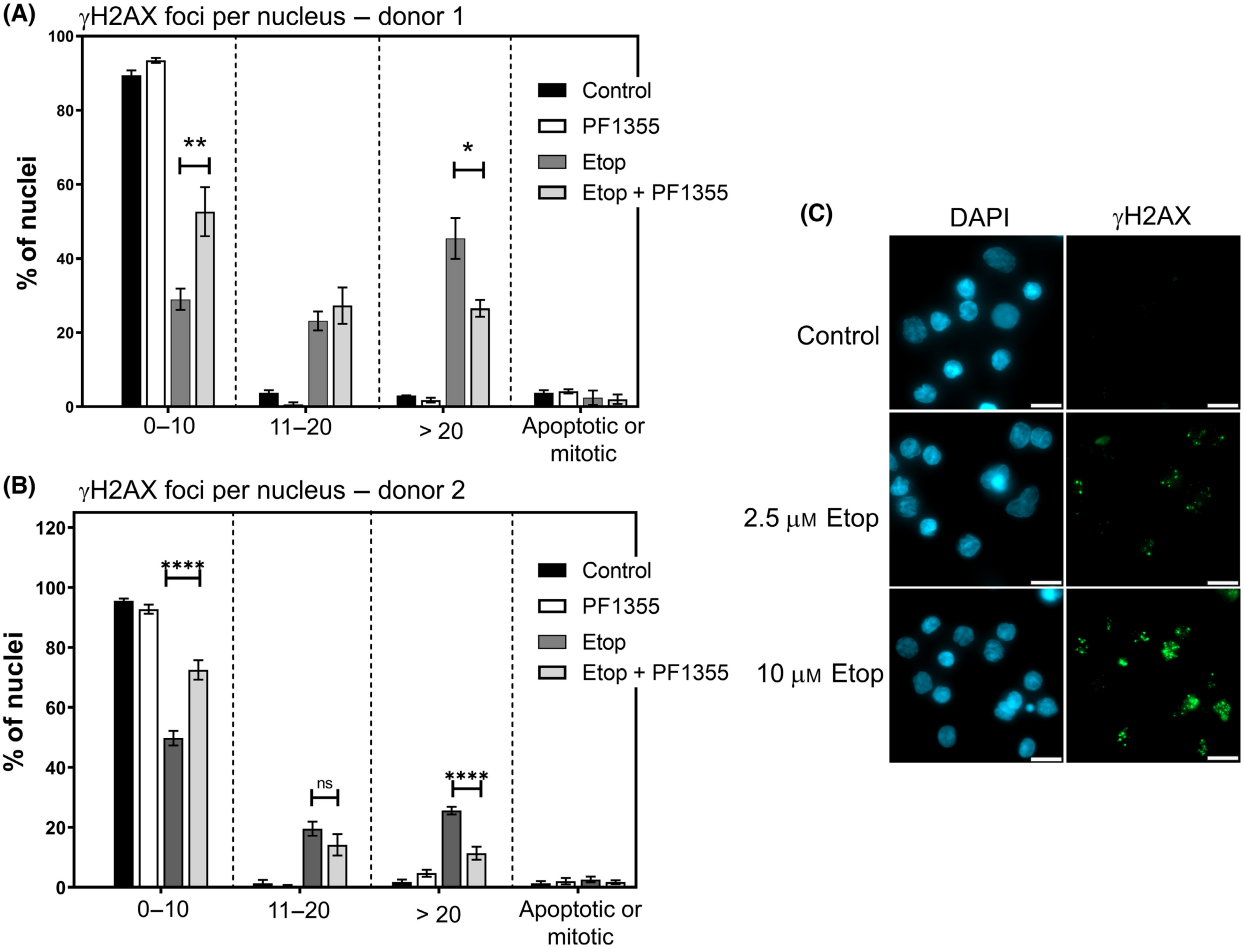
MPO inhibition suppresses etoposide‐induced DNA damage in *ex vivo* bone marrow MNCs Cryopreserved bone marrow MNCs derived from single donors were thawed and cultured overnight (18 h) in the presence or absence of PF1355 as indicated. Cells were then treated with etoposide (10 μm) or solvent control for 2 h before attaching to poly‐l‐lysine‐coated coverslips. (A, B) γH2AX immunofluorescence was performed, and cells were binned according to the number of γH2AX foci present. Data are the mean values obtained from three replicas ± SEM. Significance testing was performed using 2‐way ANOVA (Tukey; *P < 0.05, **P < 0.01, ****P < 0.0001). (C) Representative image illustrating γH2AX staining. Scale bar = 10 μm.

Treatment with 10 μm etoposide induced distinct γH2AX foci in BMCs, which were suppressed in number in cells co‐treated with PF135. After placing nuclei into categories based on foci number bins, co‐treated cells contained a larger proportion of cells with low numbers of foci (0–10 foci) and fewer cells with > 20 foci (Fig. 5A). The data shown in Fig. 5A were obtained from BMCs from a single donor, and so the experiment was repeated with BMCs from a second donor. The results of this experiment are shown in Fig. 5B. As before, co‐treatment with PF1355 shifted the distribution of foci number per cell such that fewer cells contained > 20 foci, while a greater proportion of cells contained low foci numbers (0–10). Representative images from etoposide (alone) treated cells are shown in Fig. 5C.

## Discussion

Therapy‐related acute leukaemia is a rare but very serious complication of cytotoxic chemotherapy for a previous primary cancer. Consequently, protection of the bone marrow compartment from the effects of cytotoxic chemotherapies would be very welcome if the anti‐cancer efficacy of the treatment could be maintained. MPO is expressed almost exclusively in bone marrow myeloid cells and their precursors. Although MPO is present at high levels in granulocytes, it is also found in myeloid precursor and progenitors and is readily detectable in *ex vivo* normal human bone marrow CD34^+^ cells [13]. Since MPO contributes to the conversion of TOP2 poisons including etoposide, to forms with greater DNA damaging activity, its myeloid‐specific expression may account for the apparent sensitivity of the myeloid compartment. If this is the case, MPO inhibition could partially protect myeloid cells from TOP2 poison‐mediated DNA damage. We previously demonstrated that MPO depletion or inhibition did protect the MPO‐expressing myeloid cell line NB4 from etoposide‐induced DNA damage, and that exogenous expression of MPO in the normally non‐expressing cell line K562 sensitised this line to DNA damage [13]. In the experiments described here we extend this finding to show that MPO inhibition reduces the degree of DNA damage induced by etoposide in primary *ex vivo* expanded bone marrow CD34^+^ cells and in *ex vivo* cultured unfractionated bone marrow MNCs. Since these cells consistently express TOP2B, but mostly lack significant TOP2A this effect is presumably mediated by TOP2B. Furthermore, in our previous cell line‐based study, we found that MPO activity had an approximately three‐fold greater effect on TOP2B‐CC complex formation than on TOP2A. This is significant as TOP2B appears to be required for the majority of etoposide‐induced *MLL* and *RUNX1* chromosomal breaks [10, 34, 35] in a human lymphoblastoid cell line model and for etoposide‐mediated carcinogenesis in a mouse model [36].

In addition to its relevance in the aetiology of t‐AL, MPO has become an important target for novel anti‐inflammatory drug development [23] and a number of potent specific MPO inhibitors have recently been reported, including PF1355 [24, 25], MPOi‐II [32] and AZD4831 the latter of which is currently in early clinical trials in patients with heart failure [37, 38]. Thus, in principle, unwanted TOP2 poison‐induced genotoxic damage in critical bone marrow cells could in the future be suppressed by repurposing novel compounds developed for a different clinical need.

## Conflict of interest

The authors declare no conflict of interest.

### Peer review

The peer review history for this article is available at https://www.webofscience.com/api/gateway/wos/peer‐review/10.1002/2211‐5463.13799.

## Author contributions

CAA and IGC participated in research design and wrote the paper. IGC conducted experiments and performed data analysis.

## Data Availability

The data that support the findings of this study are available from the corresponding author caroline.austin@ncl.ac.uk upon reasonable request.
